# Postweaning Isolation Rearing Alters the Adult Social, Sexual Preference and Mating Behaviors of Male CD-1 Mice

**DOI:** 10.3389/fnbeh.2019.00021

**Published:** 2019-02-28

**Authors:** Zi-Wei Liu, Yu Yu, Cong Lu, Ning Jiang, Xiao-Ping Wang, Shui-Yuan Xiao, Xin-Min Liu

**Affiliations:** ^1^Department of Psychiatry and Mental Health Institute of the Second Xiangya Hospital, Central South University, Changsha, China; ^2^National Clinical Research Center on Mental Disorders and National Technology Institute on Mental Disorders, Hunan Key Laboratory of Psychiatry and Mental Health, Changsha, China; ^3^Department of Social Medicine and Health Management, Xiangya School of Public Health, Central South University, Changsha, China; ^4^Research Center for Pharmacology and Toxicology, Institute of Medicinal Plant Development, Chinese Academy of Medical Sciences and Peking Union Medical College, Beijing, China

**Keywords:** postweaning, isolation rearing, social behavior, sexual preference, mating behavior, male CD-1 mice

## Abstract

**Objective:** No study has comprehensively evaluated the effect of postweaning isolation on the social and sexual behaviors of a certain strain of rodents in ethology. The present study aims to explore how and to what extent isolation rearing after postweaning affects the social and sexual behaviors of male CD-1 mice in adulthood systematically.

**Methods:** Male CD-1 mice were randomly assigned to two groups: isolation reared (IS, one mouse per cage, *n* = 30) and group housed (GH, five mice per cage, *n* = 15). The mice underwent isolation rearing from postnatal day 23 to day 93. Then, social affiliation, recognition and memory were measured through selection task experiments. Social interaction under a home cage and novel environment were measured via resident–intruder and social interaction test, respectively. Furthermore, sexual preference, homosexual and heterosexual behaviors were measured.

**Results:** Our study found that postweaning isolation increased the social affiliation for conspecifics (*p* = 0.001), reduced social recognition (*p* = 0.042) and impaired social memory. As for social interaction, isolated mice showed a remarkably increased aggression toward the intruder male in a home cage or novelty environment. For instance, isolated mice presented a short attack latency (*p* < 0.001), high attack frequency (*p* < 0.001) and long attack duration (*p* < 0.001). In addition, isolated mice exhibited further social avoidance. Contrastingly, isolated mice displayed a reduced sexual preference for female (IS: 61.47 ± 13.80%, GH: 70.33 ± 10.06%, *p* = 0.038). As for heterosexual behavior, isolated mice have a short mating duration (*p* = 0.002), long mounting latency (*p* = 0.002), and long intromission latency (*p* = 0.015). However, no association was observed between postweaning isolation and homosexual behavior in male CD-1 mouse.

**Conclusion:** Postweaning isolation increased the social affiliation, impaired the social cognition and considerably increased the aggression in social interaction of adult male CD-1 mice. Postweaning isolation induced a decreased sexual preference for female in adulthood. Postweaning isolation extended the latency to mate, thereby reducing mating behavior. No association was observed between isolation and homosexual behavior.

## Introduction

Early life stress can produce far-reaching deleterious effects on behavior in adult life (Lupien et al., 2009). Postweaning isolation in rodents leads to severe behavioral problems in adult life (Fone and Porkess, 2008). Studies on aberrant behavior changes in rodents resulting from stress have implications for further understanding of human behavior and psychiatric disorders. For rat and mouse, weaning can be as early as 18 days after birth. However, postnatal day (PND) 21 is often used as the beginning of postweaning.

Postweaning social isolation in rat and mouse induces behavioral changes that provide a set of models for abnormal social behaviors. Social behavior is critical for establishing and maintaining social structures in rat and mouse. In ethology, behavioral paradigms that assess social motivation/affiliation, cognition and interaction are used to elucidate the social behavior of rodents (Bicks et al., 2015). Social affiliation is often assessed via social preference tests that evaluate the time spent with a novel social target compared with the time spent on exploring a novel object or simply an empty cage (Winslow, 2003). Six weeks of postweaning isolation increase the preference of male Prairie Vole for the novel conspecific over the empty cage (Pan et al., 2009). Social cognition is a complicated concept and is difficult to measure accurately. In rodents, researchers often test social recognition or memory to reflect the social cognition function. Social recognition and memory are key aspects of normal social functioning and are considered requirements for forming long-term relationship, dominance, and other complex social behaviors in animals (Bicks et al., 2015). Social recognition and memory are measured in selection tasks with habituation/dishabituation sequences. Isolated mice from PND 30 to PND 60 exhibited a decline capability for social recognition and were less likely to discriminate between familiar and unfamiliar conspecifics (Kercmar et al., 2011). This finding indicated isolation affects the social cognition. Social interaction tests involve observing how two unfamiliar rats/mice placed together in a novel environment will explore the new cage and investigate each other (Crawley, 2007). Social interactions in rats and mice include approaching, following, sniffing, climbing onto and grooming each other (Winslow, 2003; Crawley, 2007). Isolated rats from PND 19 to PND 72 displayed marked elevations in the numbers of contacts and total time spent in contact during social interaction test (Varlinskaya and Spear, 2008; Hermes et al., 2011; Moller et al., 2013).

The strain and species generality of the effect of postweaning isolation on social behavior was studied. However, the finding was inconsistent in rodents. Six weeks of isolation increased the preference of male Prairie Vole for the chamber containing a novel male individual over the empty chamber in the social affiliation test (Pan et al., 2009). However, male Wistar rats isolated from PND 25 to PND 63 showed no difference in social affiliation compared with group-housed conspecifics (Lukasz et al., 2013). These findings indicated that the effect of isolation on social affiliation may be varied amongst strains of rodents. On the contrary, the isolated male C57BL/6J mouse from PND 30 to PND 60 presented a reduced capability for social recognition to an unfamiliar female mouse from a familiar conspecific (Kercmar et al., 2011). Male CD-1 mice isolated for 4 weeks showed reduction in social recognition toward conspecifics (Fujiwara et al., 2017). Male juvenile Swiss and C57BL/6J mice that undergone 7- and 30-day social isolation showed no impaired short-term social memory, but the long-term social memory was impaired (Gusmao et al., 2012; Monteiro et al., 2014). As for reciprocal social interaction, isolated Wistar rats, beginning at PND 22 and maintained for 2 weeks, displayed less approach and further avoidance behaviors (Hol et al., 1999). Similarly, after isolating for 3 weeks (beginning at PND21), male Sprague-Dawley (SD) rats displayed an increased latency to approach the unfamiliar conspecific and a decreased number and duration of social contacts (Lukkes et al., 2009). Male CD-1 mice isolated for 4 weeks presented a reduced social interaction, increased escape behavior and further aggressive behavior (Koike et al., 2009). However, male Mongolian gerbils isolated from PND 28 displayed marked increases in sniffing, aggression, wrestling, walking and digging in social interaction test (Shimozuru et al., 2008). These results indicated that different strains of rodents will exhibit various social behaviors after social isolation. Furthermore, postweaning isolation induced a consistent increase in aggression in rat and mouse. Isolation-reared rats exhibited further playful fights during social interaction (Han et al., 2011). Isolated mice, including C57BL/6J, NC900 and CD-1 strains, presented short attack latency, further aggressive behavior and increased biting and tail ratting times during the social interaction test (Ibi et al., 2008; Nehrenberg et al., 2010; Dang et al., 2015). In summary, the effect of postweaning isolation on social behavior may be different due to the used strain of rat or mouse.

The effect of social isolation on sexual preference has not been confirmed in rat or mouse. Sexual preference can be defined as the inclination of a rat/mouse to spend more time to interact sexually with one conspecific than with another when given the selection between an oestrous female and a sexually active male (Bakker et al., 1995). Postweaning isolated rats showed higher preference for the oestrous female than the active male (Bakker et al., 1995). However, male rats housed alone for 60 days in adulthood spent less time examining the odors of females in the odors preference test (Brown, 1985). The effect of postweaning isolation on the sexual preference of CD-1 mice remains unclear.

Sexual behavior is an aspect of sociability with highly interactive characteristics, not only a natural capability but also having some element of nurture (Hull and Dominguez, 2007). Sexual behavior in rodents includes sexual motivation, sexual exploration, sexual pursuit, copulatory mounting, copulation, and postcopulatory grooming (Green, 1966). The basic sequence consists of mating behavior, which is described as sniffing, following, mounting, intromission and ejaculation in male and lordosis posture in female. Isolation has been proven to produce sexual behavior changes in rodents. Most studies on postweaning isolation have shown that isolated rat and mouse displayed sexual behavior deficits. For instance, postweaning isolated rat displayed fewer mounts and intromissions than socially housed males in adulthood (Bakker et al., 1995). In addition, after isolation for 10–12 weeks, male rats (PND 56) showed an extension of ejaculation latency in sexual behavior compared with the double-housed control (Wallace et al., 2009). For females, C57BL/6J mice individually housed from day 25 to day 60 less often displayed lordosis postures in sexual behavior (Kercmar et al., 2014). However, one study reported the facilitation of sexual activity by isolation in Swiss, C57BL/6J and DBA mice. Isolation rearing for 2 weeks induced further mounts, intromissions and ejaculation and short latencies to the first mount and intromission (de Catanzaro and Gorzalka, 1979).

The social and sexual behaviors of rat and mouse have been investigated for numerous purposes in laboratories, ranging from theoretically oriented investigations of the causes and mechanisms of sociability and reproduction to a search for drugs to treat social or sexual dysfunction. Nonetheless, the effect of isolation during the critical period of growth on social and sexual behaviors has not been thoroughly disclosed. We hypothesized that postweaning isolation can comprehensively alter social and sexual behaviors. However, no study has systematically examined the effect of postweaning isolation on social and sexual behaviors, especially concentrating on a certain strain of rodents. Therefore, we aimed to disclose the effect of postweaning isolation rearing in CD-1 mice, due to its extensive utility in experiments and studies. In addition, we only focused on males. As the behavioral performance amongst different genders was substantially varied, the contributions of females are considered equally important. In short, the aims of the present study are: (1) to evaluate the social affiliation, recognition and interaction of the male CD-1 strain of mouse systematically after postweaning isolation, (2) to determine the sexual preference of male CD-1 mice after isolation rearing and (3) to confirm the mating behavior changes, including homosexual and heterosexual behaviors, of male CD-1 mice after isolation rearing.

## Materials and Methods

### Animals

Forty-five CD-1 mice (PND 20, Vital River, Beijing, China) were housed in black opaque polypropylene cages under standard conditions (21–25 °C, 40–60% humidity, food and water *ad libitum*, 12 h:12 h light/dark cycle). Animals were acclimatized for 3 days. Then, mice were randomly divided into two groups at 23 days of age on the basis of weight: isolation reared (one mouse per cage) and group housed (five mice per cage). The randomization process was implemented by the SAS9.2 software package. We started the experiments 10 weeks after isolation rearing. All experiments were performed in compliance with the guidelines of the Principles of Laboratory Animal Care (NIH Publication No. 80-23, revised 1996) and under the approval and supervision of the Academy of Experimental Animal Centre of the Institute of Medicinal Plant Development (China).

### Social Affiliation Test

Two types of selection task were adopted to assess social affiliation. The equipment was an open field box with black walls and floor. Two clear cylindrical cages (diameter and height of 8.5 and 11 cm, respectively) were placed in the left and right sides of the apparatus. In the first experiment, an unfamiliar male mouse was placed in a cage, whereas another cage remained empty (Winslow, 2003). Each mouse was placed in the center of the equipment and allowed to explore the arena freely for 5 min, which was recorded in a video. The total time spent near the two cylindrical cages was measured and analyzed by watching the video. The recognition index consisted of dividing the stranger exploration time by the total exploration time. In the second experiment, an unfamiliar male mouse was placed in a cage, whereas another cage contained an object. Each mouse was placed in the equipment and allowed to explore the stranger mouse and object freely for 5 min. The total time spent near the two cylindrical cages was analyzed. The recognition index also consisted of dividing the stranger exploration time by the total exploration time.

### Social Recognition Test

Social preference paradigms with habituation/dishabituation sequences were adopted to assess social recognition (Kaidanovich-Beilin et al., 2011; Kentrop et al., 2018). The apparatus used the equipment of the social affiliation test. The experimental procedure consisted of three phases: habituation with environment, social interest and social discrimination. In the habituation phase, each mouse was placed in the middle compartment and allowed to explore the arena and two empty cages freely for 5 min. In the social interest phase, an unfamiliar male mouse was placed in a cage, whereas another cage remained empty. The test mouse was placed in the apparatus again for 5 min to ensure familiarization with the stimulus mouse. In the social discrimination phase, an unfamiliar mouse was placed in another empty cage. The test mouse was placed back and allowed to explore the familiar and unfamiliar stimulus mouse for 5 min, which was recorded in a video. The times spent near each cage and near the two cages were analyzed. The discrimination index consisted of dividing the unfamiliar mouse exploration time by the total exploration time.

### Social Memory Test

The capability to recognize familiar female conspecifics was tested via a social memory test using equipment consisting of one cage within an open field box (Bielsky et al., 2004). In the test, a female stimulus mouse was placed in the cage. Each test mouse was placed in the equipment, allowed to explore the cage for 60 s and then removed. The test mouse was placed in the equipment again after 9 min, and this process was repeated three times. After a final 9 min break, a new, unfamiliar female was placed in the cage for the fifth test. During each 1 min trial, the sniffing duration of the tested mice was recorded and analyzed after completing the experiment by watching the video.

### Resident–Intruder Test

The resident–intruder test was adopted to measure the aggression of mice (Koolhaas et al., 2013). In the test, a group-reared CD-1 mouse of a similar age was placed in the home cage of each test mouse, and their behaviors were videotaped over a 10 min period. The following behaviors were analyzed: aggressive (biting, tail rattling, wrestling, and lateral threats) and non-aggressive (sniffing each other) social behaviors. The indicators were measured: attack latency, attack frequency and duration, upright posture and unaggressive social contact.

### Social Interaction Test

The social interaction test was adopted to measure deficits in reciprocal interaction behavior (Berry et al., 2012). The test was conducted in the open-filed box of black Plexiglas with a transparent wall in front, under a novel environment for the test mouse. On the day of testing, test and stimulus mice were placed simultaneously into the apparatus for 10 min. The stimulus mouse was marked by a yellow, scentless and non-toxic paint before testing to discriminate the experimental subject from the stimulus conspecific during data collection. The frequency and duration of environmental exploration, social interaction (body, nose and anogenital sniffing and exploration by stimulus mouse), non-social behaviors (self-grooming, motionlessness, upright posture and rest) and aggressive behaviors (latency to attack and attack frequency and duration) were determined (Hamilton et al., 2014).

### Sexual Preference Test

The social preference paradigm was adopted to measure the sexual preference in mice (Winslow, 2003). Isolation-reared male mice and control group were sexually inexperienced. A three-chambered box (each chamber: 40 cm × 40 cm × 40 cm) was used as the test apparatus (Zhang et al., 2013). A CD-1 sexually experienced and active male (aging 13 weeks) and an oestrous CD-1 female were placed on each side the chamber. A cylindrical wire mesh cage (diameter and height of 8.5 and 11 cm, respectively) was used to prevent the experimental animal from having physical contact with the stimulus mice. The testing period lasted for 15 min. The time spent for exploring female and male stimulus mice was recorded. The sexual preference index was calculated by dividing the time spent for exploring the female to the total time spent for exploring the female and male (Zinck and Lima, 2013).

### Homosexual Behavior Test

Three days after the sexual preference test, isolation-reared mice and the control group, still sexually inexperienced, were tested for homosexual behavior. The day before the test, each mouse was placed singly into the test apparatus and allowed to habituate it for 30 min. During testing, the test mouse habituated the test box (40 cm × 40 cm × 40 cm) for 10 min, after which a sexually active and experienced male was placed in the box. The testing period lasted 30 min. Anogenital sniffing duration, latencies to first mount and intromission, total numbers of mounts, intromissions and ejaculations and lordosis were recorded (Haga et al., 2010). Lordosis was defined as a male exhibiting the sexual receptivity of a female with four paws grounded, the hind region elevated from the floor of the test chamber, with no evidence of attempt to escape or exhibit a defensive upright posture, and the back slightly arched (Kudwa et al., 2005).

### Heterosexual Behavior Test

Three days after the homosexual behavior test, all mice were used in heterosexual behavior test (Siegel et al., 1981; Sisk and Meek, 2001). The test box used in the homosexual behavior test was utilized. An oestrous female was prepared by injecting 20 μg of oestradiol benzoate and 500 μg of progesterone into an ovariectomised female 48 and 4 h, respectively, prior to testing. During testing, the test mouse was placed in the test apparatus and allowed to habituate it for 10 min. Then, an oestrous female (postnatal 13 weeks) was placed in the test box. The testing period lasted 30 min. Latencies to first mount, intromission and ejaculation, refractory period and frequencies and durations of mounts, intromissions and ejaculations were recorded.

### Statistical Analyses

Data were analyzed using the SPSS 22.0 software package. Comparisons of parametric data were performed using Student’s *t*-test and the Chi-squared test for two groups. For non-parametric data, the Mann–Whitney *U*-test was used for two groups.

## Results

### Social Affiliation

**FIGURE 1:**
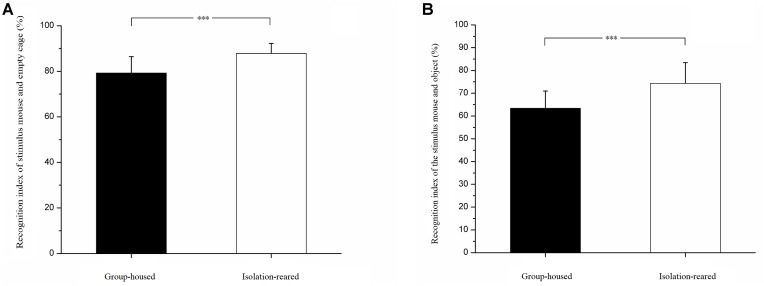
Results of social affiliation test. **(A)** Recognition indexes of stimulus mouse and empty cage. **(B)** Recognition indexes of stimulus mouse and object. ^∗∗∗^ indicate *p* < 0.001. Bar represent mean. Error bar represent standard deviation (SD).

In the selection task involving a stimulus mouse and empty cage, isolation-reared mice spent more time exploring stimulus mice than did group-housed mice (IS: 7.43 ± 3.74, GH: 0.40 ± 4.02, *p* = 0.019). Isolation-reared mice spent less time exploring the empty cage than did group-housed mice (IS: 49.28 ± 14.54, GH: 37.81 ± 15.32, *p* = 0.022). The recognition index was higher in isolation-reared mice than in group-housed mice (IS: 87.85% ± 4.40%, GH: 79.25% ± 7.19%, *p* = 0.001) (Figure 1A), which indicates that postweaning isolation rearing increases the CD-1 mouse social affiliation for conspecifics.

In the selection task involving a stimulus mouse and object, isolation-reared mice spent more time exploring stimulus mice than did group-housed mice (IS: 55.66 ± 18.13, GH: 33.46 ± 13.66, *p* < 0.001). However, isolation-reared and group-house mice spent similar times exploring objects (IS: 19.08 ± 7.83, GH: 18.15 ± 4.66, *p* = 0.624). The recognition index was higher in isolation-reared mice than in group-housed mice (IS: 74.34% ± 9.10%, GH: 63.36% ± 7.61%, *p* < 0.001) (Figure 1B), which indicates that postweaning isolation rearing increases the male CD-1 mouse social affiliation for conspecifics.

### Social Recognition

**FIGURE 2:**
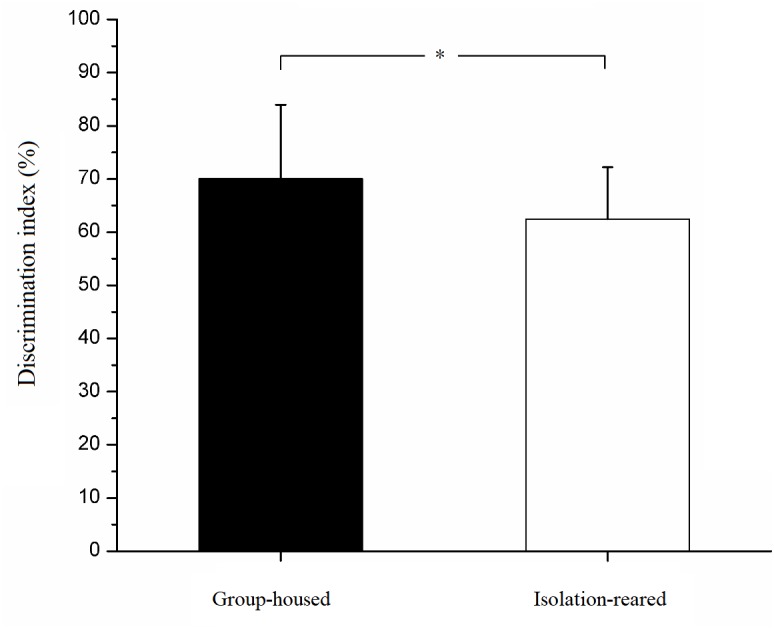
Discrimination index of isolation-reared and group-housed mice in the social recognition tests. ^∗^Indicate *p* < 0.05. Bar represent mean. Error bar represent SD.

In the habituation phase, both isolation-reared and group-housed mice displayed no difference in their exploration times of two unfamiliar male mice. In the testing phase, isolation-reared mice spent more time exploring familiar male mice than did group-housed mice (IS: 24.71 ± 10.74, GH: 12.67 ± 7.15, *p* < 0.001). In addition, isolation-reared mice spent more time exploring unfamiliar male mice than did group-housed mice (IS: 39.74 ± 12.41, GH: 23.93 ± 10.89, *p* < 0.001). However, the discrimination index was lower in isolation-reared mice than in group-housed mice (IS: 62.45 ± 9.77, GH: 70.06 ± 13.92, *p* = 0.042) (Figure 2), which indicates that postweaning isolation rearing reduces the ability of male CD-1 mice to recognize familiar male conspecifics.

### Social Memory

**FIGURE 3:**
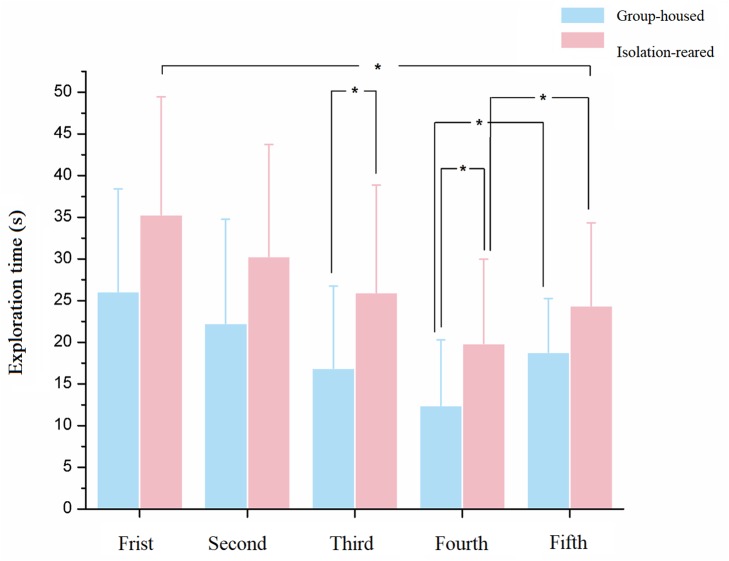
The exploration time of isolation-reared and group-housed mice in the social memory tests. ^∗^Indicate *p* < 0.05. Bar represent mean. Error bar represent SD.

All mice were exposed to the same female stimulus mouse thrice for 60 s each time as the first three tests and then introduced to a new female stimulus mouse as the fourth test. The time spent exploring declined from the first to the fourth tests (*p* = 0.050, 0.077, 0.030, and 0.025, respectively). The exploration time recovered in the fifth test (test with a new female, *p* = 0.071). Exploration times recovered to levels observed in the first test during the fifth test in group-housed mice but not in isolation-reared mice, which indicates that postweaning reduces the ability of male CD-1 mice to memorize familiar female conspecifics and recognize a new female (Figure 3).

### Aggression

**FIGURE 4:**
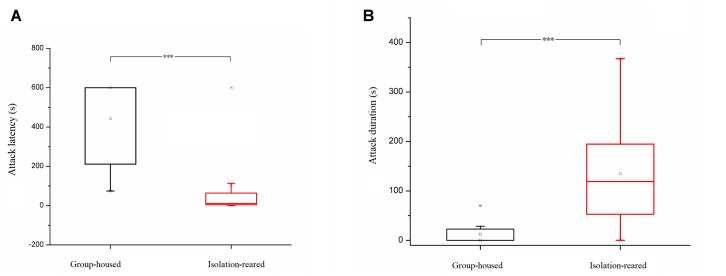
Box plots of attack latency and duration in the resident–intruder tests. **(A)** Attack latency of isolation-reared and group-housed mice. **(B)** Attack duration of isolation-reared and group-housed mice. ^∗∗∗^Indicate *p* < 0.001. Bar represent minimum or maximum. ^□^Represent mean.

In the resident–intruder test, isolation-reared mice displayed a shorter latency to attack (*p* < 0.001) (Figure 4A), higher attack frequency (IS: 46, GH: 0, *p* < 0.001), longer attack duration (*p* < 0.001) and increased frequency of upright posture (IS: 1, GH: 0, *p* = 0.003) (Figure 4B) compared with group-housed mice. However, non-aggressive social contact times and durations were not different between the isolation-reared and group-housed mice (IS: 20, GH: 16, *p* = 0.495 and IS: 100.99 s, GH: 176.03 s, *p* = 0.077, respectively). These results indicate that postweaning increases aggression in male CD-1 mice.

### Reciprocal Social Interaction

Compared with the control mice, isolated mice displayed significantly increased aggression and rejection of contact from the stimulus mouse in the social interaction test within a novel environment. However, no differences in terms of social contact with the stimulus mouse and self-grooming were observed between the isolated and group-housed mice during environment exploration. Specifically, isolated mice displayed a higher incidence of attack (IS: 96.6%, GH: 7.1%, *p* < 0.001), shorter attack latency (*p* < 0.001), higher frequency of attack (*p* < 0.001), and longer attack duration (*p* < 0.001) than did group-housed mice. Isolated mice also showed a short duration of environment exploration (*p* = 0.002). No difference in the frequency of environment exploration was observed between groups (*p* = 0.459) (Table 1).

**Table 1:** Social interaction of isolation-reared and group-housed mice.

	Isolation-reared	Group-housed	*Z*	*p*
Frequency of environment exploration	30 (12.5)	26.5 (3.0)	-0.74	0.459
Duration of environment exploration	347.18 (164.03)	452.11 (122.6)	-3.16	0.002
Frequency of stimulus mouse exploration	22 (20)	23 (9.25)	-0.79	0.428
Duration of stimulus mouse exploration	96.72(160.97)	98.00 (54.80)	-0.18	0.856
Attack latency	20.19 (40.72)	600.00 (0.00)	-5.08	<0.001
Frequency of attack	45 (41.5)	0 (0)	-4.84	<0.001
Duration of attack	122.94 (113.78)	0.00 (0.00)	-4.71	<0.001
Frequency of self- grooming	2(1)	2(2)	-0.52	0.604
Duration of self-grooming	10.75 (5.86)	15.33 (19.08)	-0.96	0.337
Frequency of rest	0(1)	0(2)	-0.36	0.722
Duration of rest	0.00(4.59)	0.00(1.53)	-0.46	0.648
Frequency of upright	53 (33.5)	82.5 (32.25)	-3.51	<0.001
Frequency of tail-rattling	1(2)	0(0)	-3.20	0.001
Duration of tail-rattling	1.10 (4.65)	0.00(0.00)	-3.20	0.001
Frequency of contacting from stimulus mouse	0.00 (0.00)	3.5(4.75)	-5.20	<0.001
Duration of contacting from stimulus mouse	0.00 (0.00)	31.49(56.02)	-5.44	<0.001


*Median (Interquartile range, IQR).*

In the test of social contact with the stimulus mouse, no differences in the frequency and duration of exploration of the stimulus mouse (*p* = 0.428 and 0.856) were observed between the isolated and group-housed mice. In addition, isolated mice showed lower frequencies and durations of rejection during contact by the stimulus mouse (both *p* < 0.001) compared with group-housed mice.

### Sexual Preference

**FIGURE 5:**
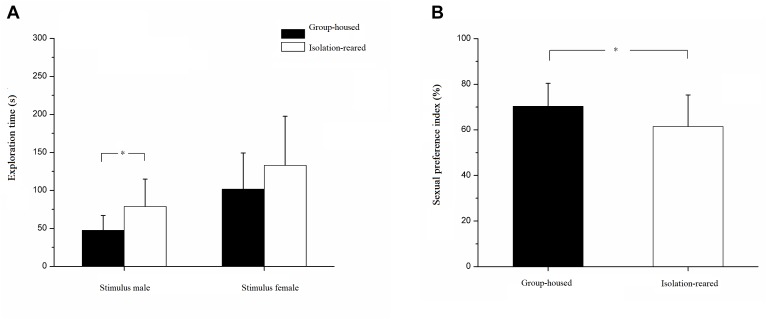
Results of sexual preference test. **(A)** The exploration time of isolation-reared and group-housed mice toward stimulus male and female. **(B)** The sexual preference indexes of isolation-reared and group-housed mice. ^∗^Indicate *p* < 0.05. Bar represent mean. Error bar represent SD.

Our results illustrate that isolated mice spent more time exploring male mice than did group-housed mice (IS: 78.70 ± 36.12 s, GH: 47.23 ± 19.65 s, *p* = 0.003). However, the time spent exploring the female mouse did not differ between the isolated and group-housed mice (IS: 132.79 ± 64.65 s, GH: 101.44 ± 47.82 s, *p* = 0.114) (Figure 5A). The sexual preference index was significantly lower in isolation-reared mice than in group-housed mice (IS: 61.47 ± 13.80, GH: 70.33 ± 10.06, *p* = 0.038) (Figure 5B), thereby implying that postweaning isolation may lead to decreased interest in females amongst male CD-1 mice.

### Homosexual Behavior

No sexual behaviors, included mounting, intromission and ejaculation, were observed between the test mice and stimulus males. Isolated mice showed decreases in anogenital sniffing frequency and shorter anogenital sniffing durations (*p* = 0.003 and 0.008, respectively) compared with group-housed conspecific mice (Table 2).

**Table 2:** Homosexual behavior of isolation-reared and group-housed mice.

	Isolation-reared	Group-housed	*Z*	*p*
Anogenital sniffing frequency	0 (2)	4 (4)	-2.98	0.003
Anogenital sniffing duration	0.00 (1.92)	5.39 (6.25)	-2.64	0.008


*Median (IQR).*

### Heterosexual Behavior

**FIGURE 6:**
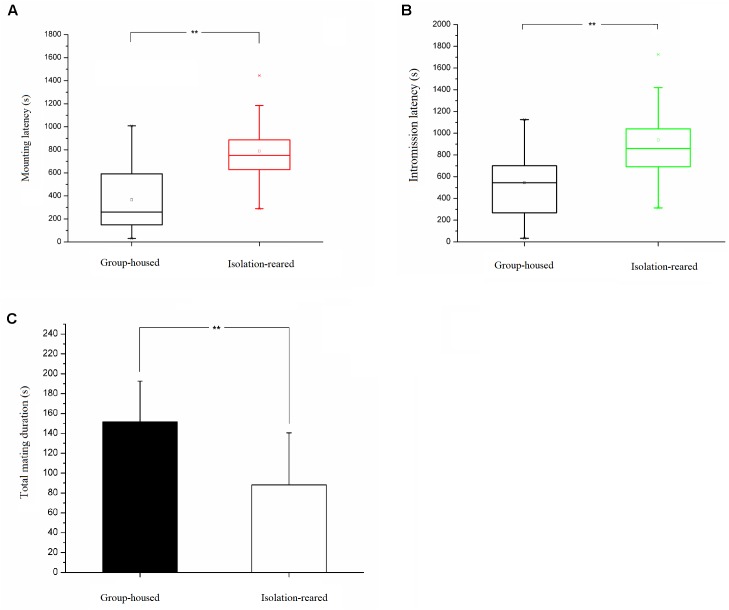
Results of heterosexual behavior test. **(A)** Box plot of the mounting latency of isolation-reared and group-housed mice. **(B)** Box plot of the intromission latency of isolation-reared and group-housed mice. **(C)** The total mating duration of isolation-reared and group-housed mice. ^∗∗^Indicate *p* < 0.01.

The mating success rate tended to be lower in the isolation group than in the group-housed group (IS: 80.0%, GH: 86.7%), although no statistically significant difference between groups was observed (*p* = 0.458). The latency to first mount was longer in the isolation group than in the group-housed group (*p* = 0.002) (Figure 6A), which means the former required a longer time to enter into a sexual procedure than did the latter. The latency to first intromission was longer in the isolation group than in the group-housed group (*p* = 0.015) (Figure 6B), which indicates that the former required a longer time to attract female mice than did the latter group. No statistically significant difference between the two groups was observed in terms of latency to ejaculation and refractory period. The total mating duration was shorter in the isolation group (Table 3) than in the group-housed group, and differences observed were statistically significant (*p* = 0.002) (Figure 6C). No statistically significant differences between the two groups were observed in terms of frequency of mounting before ejaculation, total frequency of mounting, number of intromissions and total number of mating (Table 4).

**Table 3:** Heterosexual behavior of isolation-reared and group-housed mice.

	Isolation-reared	Group-housed	*t/t’*	*p*
Mounting latency	788.70 ± 262.77	365.03 ± 288.65	-3.87	0.002
Intromission latency	937.30 ± 369.87	542.94 ± 352.40	-2.75	0.015
Ejaculation latency	16.58 ± 9.78	17.37 ± 13.03	-0.20	0.845
Refractory period	173.00 ± 89.84	192.87 ± 106.91	0.58	0.565
Total mating duration	88.27 ± 52.40	151.65 ± 40.87	3.44	0.002


*Mean ± SD.*

**Table 4:** Results of heterosexual behavior test.

	Isolation-reared	Group-housed	*Z*	*p*
Number of mounting before ejaculation	2 (2)	2 (3)	-0.77	0.442
Number of intromissions	18 (19)	18 (21)	-0.28	0.779
Total number of mounting	11 (7.75)	18 (10.5)	-1.26	0.207
Total number of mating	7 (5.5)	10 (8)	-1.47	0.142


*Median (IQR).*

## Discussion

### Main Finding

Postweaning isolation comprehensively altered the adult social affiliation, social recognition and social interaction behaviors of male CD-1 mice. In the social affiliation test, isolated mice spent more time exploring conspecifics than the empty cage or object. In the social recognition and memory test, isolated mice displayed lower discrimination indices in exploring novel males and female, which indicates a reduced ability to discriminate a novelty mouse from a familiar one. In the resident–intruder test, isolated mice showed remarkably increased aggression toward the intruder male in their home cage. In the social interaction test, isolated mice also exhibited abnormal attack behaviors toward male conspecifics in a novel environment. In the sexual preference test, isolated mice displayed a reduced sexual preference for females. In the homosexual behavior test, no association was observed between postweaning isolation and homosexuality in male CD-1 mice. In the heterosexual behavior test, isolated mice revealed a shorter mating duration, longer latency to mount and longer latency to intromission than did group-housed mice.

### Postweaning Isolation Altered Adult Social Affiliation, Social Cognition and Social Interaction in Male CD-1 Mice

Our results reveal that isolated mice display increased affiliation for a stimulus mouse in the social affiliation tests, decreased recognition for a novel male in the social recognition test, reduced memory for a familiar female in the social memory test and increased aggression in the social interaction test. CD-1 mice are social animals, and their typical group social structure has been widely researched for many purposes. Several ethological experiments have been designed to measure social behavior in rodents. However, past studies usually used only one or two methods to detect one or two types of social behaviors, and no study systematically examining the effect of postweaning isolation on the social ability and behavior of a certain strain of rats or mice has yet been published.

Firstly, selection task paradigms have been adopted to measure social affiliations in rodents. One kind of experimental design involves observation of a test mouse choosing between an empty cage and a cage containing a stimulus conspecific. In some cases, the experimenter places an object in the empty cage and observes the rodent’s choice making between an object and a conspecific to measure social affiliation. In the present study, isolated CD-1 mice spent more time near the cage containing a stimulus mouse than did group-housed mice, which is consistent with findings involving isolated male prairie voles (Pan et al., 2009). In addition, we found that isolated CD-1 mice demonstrate a higher affiliation for a conspecific than do group-housed mice in the choice between an object and a conspecific. This finding indicates that postweaning isolation increases the social affiliation for conspecifics in male CD-1 mouse.

Secondly, selection tasks with habituation/dishabituation sequences have been adopted to measure social recognition or memory in rodents. We found that postweaning isolation reduces the ability of male CD-1 mice to recognize a novel male, memorize familiar females, and recognize a new female. Our finding is consistent with Fujiwara’s study on CD-1 mice (Fujiwara et al., 2017) and previous work on C57BL/6J mice but not Swiss mice (Kercmar et al., 2011; Gusmao et al., 2012). Nevertheless, all evidence indicates that postweaning isolation impairs social cognition in mice.

Thirdly, in the resident–intruder and novel environment social interaction tests, compared with group-housed mice, isolated male CD-1 mice showed significantly increased aggression toward male conspecifics with a shorter attack latency, more attack behaviors (e.g., biting, tail-rattling, upright) and extended attack duration. Most strains of rats and mice show increased aggression during social interaction after postweaning isolation (Pinna et al., 2004; Ibi et al., 2008; Toth et al., 2011). We also observed that, compared with group-housed mice, isolated mice show remarkably enhanced avoidance behaviors during social interaction by rejecting contact from the stimulus mice. Thus, postweaning isolation increases social affiliation with conspecifics, impairs social cognition, and completely destroys social function in male CD-1 mice.

### Postweaning Isolation Reduced Sexual Preference in Male CD-1 Mice

Our study found that postweaning isolated mice display a lower sexual preference index compared with that of group-housed mouse. The sexual preference test utilizes animals’ interest and natural exploration of individuals of the opposite sex and determines changes in an animal’s sexual preference for the opposite sex by measuring the times they spend exploring conspecifics of the same and opposite sex (Zhang et al., 2013). In most experiments, a sexually active male rat/mouse and an oestrous female are placed on both sides of a three-chamber test box and covered with a metal wire cage to avoid running and physical contact with the target mouse (Winslow, 2003). The sexual preference index is then calculated to detect changes in animal sexual preference by measuring the times of non-contact exploration between the test animal and conspecifics of the same and opposite sex.

In this study, the sexual preference index of the isolated male CD-1 mice was lower than that of the group-housed mice, indicating a decrease in female exploration time in the former. In terms of exploration time, the average exploration time of isolation-reared mice was higher than that of the group-housed mice, although the difference observed between groups was not statistically significant. Isolation-reared mice spent more time exploring their male counterparts than did group-housed mice, and the difference found was statistically significant. Thus, the decreased sexual preference index of isolated mice is due to the increased time spent exploring males rather than the decreased time spent exploring females. The sexual preference index of isolated mice was higher than 50%, which indicates that these mice spent more time exploring females than males. Therefore, isolation rearing does not reduce the interest of male mice in exploring oestrous females but increases their interest in exploring their male counterparts, leading to a decrease in their sexual preference index.

### Postweaning Isolation Did Not Induce Homosexual Behaviors in Male CD-1 Mice

The results of the sexual preference test suggest that the isolated mice are more interested in exploring males than are group-housed mice. Therefore, we used the homosexual behavior test to detect homosexual behavior in isolation-reared and group-housed mice. No unified method to detect the homosexual behavior of rodents has yet been reported. In the experiments, we replaced the oestrous female with a sexually experienced male at a sexually active age according to the test method of heterosexual behavior (Crawley, 2007). The interaction between the test mice and their sexually active male counterparts was used to observe whether the former exhibited homosexual behaviors. The observational indicators included male homosexual behaviors, such as anogenital sniffing, mounting, intromission, ejaculation, and assumption of the posture of female sexual receptivity, also known as lordosis. The results showed that neither of the groups exhibit any specific homosexual behavior, such as intromission or ejaculation. No mounting between the test mice and the stimulus mice or lordosis was observed. However, the isolation-reared mice showed shorter anogenital sniffing durations than did the control group, indicating decreased interest in mating with the male mice. Moreover, isolation-reared mice displayed strong aggression toward males and refused contact from other male mice in the social interaction tests. This finding suggests that the observed increased exploration interest of the test mice for mice of the same sex may be due to hostility and aggression toward the same sex rather than sexual interest.

### Postweaning Isolation Extended the Latency to Mate, Leading to Reduced Mating Behaviors in Male CD-1 Mice

Our study found that isolated mice tend to have a lower mating success rate compared with group-housed mice. In addition, the former had shorter mating durations than the latter. These results indicate that postweaning isolation reduces the heterosexual behaviors of male CD-1 mice. However, isolated mice demonstrated longer latencies for mounting and intromission. An increase in latency to mount means an increase in time from the first meeting to the first mount, whilst increased latency to intromission means a longer time interval from meeting to intromission. The findings reveal that isolated male mice need more time to identify, judge and perform the mating process compared with group-housed mice. No significant difference was observed between the two groups in terms of latency to ejaculation and refractory period, which may imply that the sexual physiological function of male mice is not affected by isolation. In addition, no statistically significant difference was observed between the two groups in terms of intromission frequency, latency to ejaculation, and refractory period, which also indicates that sexual function is not affected by isolation. However, sexual behavior is not only simply a physical activity; it is also a social activity closely related to social function. Isolation rearing induces extended latencies to mount and intromission. During this latency, the interaction between a male and a female influence when the mating process should begin. Whilst increases in mating latency seem to be caused by impaired social function in isolated mice, the related mechanism remains unclear and requires further study. Taken together, the results indicate that postweaning isolation extends the latency to mate and further leads to reduced mating behaviors in male CD-1 mice.

One advantage of this study is that we systematically evaluated the effect of postweaning isolation on adult social behaviors in terms of social affiliation, social recognition, social memory and social interaction in male CD-1 mice. Another advantage is that we evaluated the sexual preference and homosexual and heterosexual behaviors of male CD-1 mice in adulthood, all of which are closely related to social function. We believe our findings add some knowledge to the body of research on isolation-induced social and sexual behavior disorders in CD-1 mice.

This study presents some limitations that must be acknowledged. Firstly, we only tested the first sexual behavior of mice and did not observe dynamic changes in sex preference and sexual behavior continuously. Secondly, the results of the current study on the association between isolation rearing and sexual behavior changes in mice may not be generalized to include humans. Nevertheless, we believe our findings are of great significance to future explorations on etiology between loss of social relationship during crucial development periods and social and sexual behavior changes in adulthood.

## Conclusion

Postweaning isolation increased the social affiliation of adult male CD-1 mice for conspecifics, impaired their social recognition and destroyed their social function during social interaction in both the home cage and a novel environment. Postweaning isolation also induced decreased sexual preference for females in the CD-1 mice and extended their latency to mate, leading to reduced mating behaviors. No association was observed between postweaning isolation and homosexual sex in male CD-1 mice.

## Data Availability

The raw data supporting the conclusions of this manuscript will be made available by the authors, without undue reservation, to any qualified researcher.

## Author Contributions

S-YX and X-ML contributed in the experimental design. X-PW contributed in the technical development. Z-WL, CL, and NJ performed the experiments. Z-WL analyzed the data and wrote the manuscript. YY helped perform the data analysis and worked over the first draft of the manuscript. All authors approved the final version of the manuscript.

## Conflict of Interest Statement

The authors declare that the research was conducted in the absence of any commercial or financial relationships that could be construed as a potential conflict of interest.
